# Inverse Association between the Omega-3 Index and Neutrophil–Lymphocyte Ratio: Pooled Results from Four Supplementation Trials

**DOI:** 10.1016/j.tjnut.2026.101663

**Published:** 2026-06-10

**Authors:** Michael I McBurney, Nathan L Tintle, Jason Westra, Mélanie Plourde, Michael Flock, David M Mutch, Charles B Stephensen, John W Newman, William S Harris

**Affiliations:** 1 Department of Human Health Sciences, University of Guelph, Guelph, ON, Canada; 2 Fatty Acid Research Institute, Sioux Falls, SD, United States; 3 Department of Population Health Nursing Science, University of Illinois, Chicago, IL, United States; 4 Département de Médecine, Université de Sherbrooke, and Research Centre on Aging, Sherbrooke, QC, Canada; 5 Office of Innovation & Entrepreneurship, University of Pittsburgh, Pittsburgh, PA, United States; 6 US Department of Agriculture, Western Human Nutrition Research Center, Davis, CA, United States; 7 Department of Nutrition, University of California Davis, Davis, CA, United States; 8 Sanford School of Medicine, University of South Dakota, Sioux Falls, SD, United States

**Keywords:** EPA, DHA, lymphocytes, neutrophils, red blood cells

## Abstract

**Background:**

The Omega-3 Index [O3I, erythrocyte eicosapentaenoic acid (EPA), and docosahexaenoic acid (DHA) content] has been inversely correlated with neutrophil–lymphocyte ratio (NLR) and red blood cell distribution width (RDW) in observational studies.

**Objectives:**

We evaluated the effects of omega-3 interventions (EPA+DHA) on O3I, NLR, and RDW values in healthy adults and tested the hypothesis that changes in O3I (ΔO3I) modified NLR and, separately, RDW.

**Methods:**

We performed a de novo, individual-level analysis of baseline (b) and post (p) intervention O3I, NLR, and RDW values from 3 randomized, placebo-controlled trials and 1 ω-3 supplementation trial, followed by an equivalent duration washout (combined *n* =436). Primary outcome effect estimates were obtained using linear mixed effects models with a statistical significance threshold set at 0.05.

**Results:**

EPA + DHA supplementation (mean dose = 1160 mg/d) increased O3I (ΔO3I = 3.28%, *P* < 0.001, *n* = 253), whereas O3I decreased (ΔO3I = –0.62, *P* < 0.001, *n* = 183) in the CONTROL group. NLR decreased in individuals treated with EPA + DHA (ΔNLR = –0.15, *P* < 0.001) compared with CONTROL (–0.02, 95% confidence interval: –0.10, 0.06), a statistically significant difference (*P* < 0.05). In contrast, EPA+DHA supplementation was not associated with changes in RDW (*P* = 0.62), whereas RDW increased by ∼1% in CONTROL individuals (ΔRDW = 0.09, *P* < 0.01). ΔNLR values were inversely correlated with ΔO3I in unadjusted (model 1, *r* = –0.08, *P* < 0.05) and models that adjusted for baseline NLR, age, and sex (model 2, *r* = –0.07, *P* < 0.05). There was no evidence of an association between ΔO3I and ΔRDW (model 2, *r* = 0.003, *P* = 0.37).

**Conclusions:**

In healthy adults, EPA+DHA increased O3I and reduced NLR. ΔO3I was inversely correlated with ΔNLR but not with ΔRDW. Further clinical trials are needed to determine if ω-3 supplementation causes biologically relevant changes in NLR, and possibly RDW, to affect clinical outcomes in individuals with elevated NLR and RDW, and their associated increased risk of morbidity and mortality.

## Introduction

The physical properties of blood cells must enable them to squeeze through capillaries to reach tissues where immune cells, for example, neutrophils (N) and lymphocytes (L), protect the host, and red blood cells (RBCs) exchange gases. In intervention studies, supplemental long-chain ω-3 fatty acids increased RBC membrane proportions of EPA and DHA [Omega-3 Index (O3I)] [1], changed the physical properties of blood cells, for example, enhanced leukocyte deformability when subjected to physical stress [2], and increased RBC fluidity [3]. Observational studies report inverse correlations of O3I with the neutrophil–lymphocyte ratio (NLR) [4,5] and the RBC distribution width (RDW) [5,6], but it is unknown whether ω-3 interventions, which change in O3I (ΔO3I), would also impact NLR or RDW.

A complete blood count (CBC) quantifies cell types and CBC-derived prognostic biomarkers such as the NLR and RDW [7]. Neutrophils secrete proinflammatory mediators, and lymphocytes regulate the inflammatory response through T-regulatory lymphocytes [8]. NLR reflects the balance between innate (neutrophils) and adaptive (lymphocytes) immune responses [9,10]. Although acute inflammation protects against injury, low-grade chronic inflammation increases morbidity [11], and an elevated NLR is a strong predictor of risk of death [10,12,13]. RDW is also a biomarker of systemic inflammation and of abnormal erythrocyte function [14]. Elevated RDW values are indicative of inflammation, oxidative stress, and RBC frailty [15,16]; are used to diagnose anemia; and are associated with age-related changes in risk of morbidity and mortality [17,18]. EPA and DHA have anti-inflammatory properties [19], and studies consistently report inverse associations between intake or status of EPA and DHA with markers of inflammation [[20], [21], [22]].

In free-living, apparently healthy individuals, an inverse relationship was found between NLR and blood EPA+DHA levels up until O3I >6.6% [4]. In the United Kingdom Biobank, there was a 0.7 difference in mean NLR (1.7–2.4) observed across the O3I range [5]. This difference in NLR values corresponded to an estimated 24% increased risk of all-cause mortality when moving from the highest O3I quintile (>6.89%) to the lowest (<4.01%). Similarly, observational studies suggest that an O3I >5.6% may be required to maintain normal RBC structural and functional integrity [6]. More recently, studies involving multiple public databases have reported inverse associations between dietary and blood levels of ω-3 fatty acids and RDW in patients after myocardial events, consistent with the hypothesis that a higher O3I may attenuate inflammation and improve erythrocyte function [23]. However, observational studies do not establish causality. Controlled intervention trials are needed to determine if NLR or RDW values directly respond to manipulations in blood EPA+DHA concentrations.

## Objectives

We hypothesized that EPA+DHA supplementation would increase the O3I and decrease NLR and RDW, and separately, that any ΔO3I (positive or negative) would be inversely related to ΔNLR and ΔRDW. To assess these hypotheses, we combined individual-level data from 4 ω-3 clinical intervention trials with baseline (b) and postintervention (p) measurements of O3I, NLR, and RDW values (Table 1).

**TABLE 1:** Description of 4 ω-3 supplementation clinical trials.

Study	n	Sex (M/F)	Duration (wk)	Intervention (mg, EPA+DHA/d)	ω-3 analysis^1^
Pennsylvania [24]	23	11/12	20	CONTROL (placebo)	RBC, GC
	23	11/12		300	
	22	12/10		600	
	24	13/11		900	
	23	13/10		1800	
Quebec [25]	92	33/59	24	CONTROL (placebo)	PL, GC
	92	30/62		2500	
California [26,27]	45	14/31	6	CONTROL (placebo)	RBC, GC
	45	13/32		3000	
Ontario (B. MacIntyre, M. Sterling, A. Jenkins, D.M. Mutch, unpublished results)	24	12/12	8	2400	RBC, GC
	23^2^	12/11		CONTROL (washout)	

Abbreviations: PL, plasma phospholipids; RBC, red blood cell.

1 Blood sample and analytical method used in ω-3 fatty acid analysis.

2 Of the 24 individuals who completed the EPA+DHA intervention, 23 individuals returned for blood sampling following an 8-wk washout.

## Methods

### Study design, treatment assignments, and participants

Data were collated from 4 controlled ω-3 supplementation trials involving healthy males and females with baseline (b) and post (p) intervention O3I, NLR, and RDW measurements; 3 of which have been previously reported in the literature (Table 1). The Pennsylvania study is a placebo-controlled, double-blind, parallel-group study where 125 healthy adults (20–45 y) were randomly assigned to 0, 300, 600, 900, or 1800 mg EPA+DHA/d for 5 mo to model O3I responses to supplemental ω-3 intake [24]. The Quebec study is a placebo-controlled trial where 236 healthy adults (20–80 y) were randomly assigned to 0 or 2500 mg EPA+DHA/d for 6 mo to determine effects on cognitive performance [25]. The California study randomized 116 African American adults (20–59 y) to 0 or 3000 mg EPA+DHA/d for 1.5 mo to determine the effect of ω-3 supplementation on cardiovascular disease risk factors in individuals with different 5-lipoxygenase enzyme gene (*Alox5)* variants [26,27]. The Ontario study was an investigation of 24 healthy adults (19–30 y) who consumed 2400 mg EPA+DHA/d for 8 wk, followed by an 8-wk washout to investigate the effects on urinary biomarkers and to correlate these urinary metabolites with O3I (B. MacIntyre, M. Sterling, A. Jenkins, D.M. Mutch, unpublished results). For the Ontario study, blood was drawn at baseline, after ω-3 supplementation, and after the washout. Thus, O3I, NLR, and RDW values measured after 8 wk of EPA+DHA also served as baseline values (CONTROL) for the 8 wk washout. The pooled data provided sufficient statistical power (>80%) to detect small-to-medium effect sizes when comparing groups (*d* = –0.26), and small correlations (*r* = 0.13).

### Data acquisition

**FIGURE 1:**
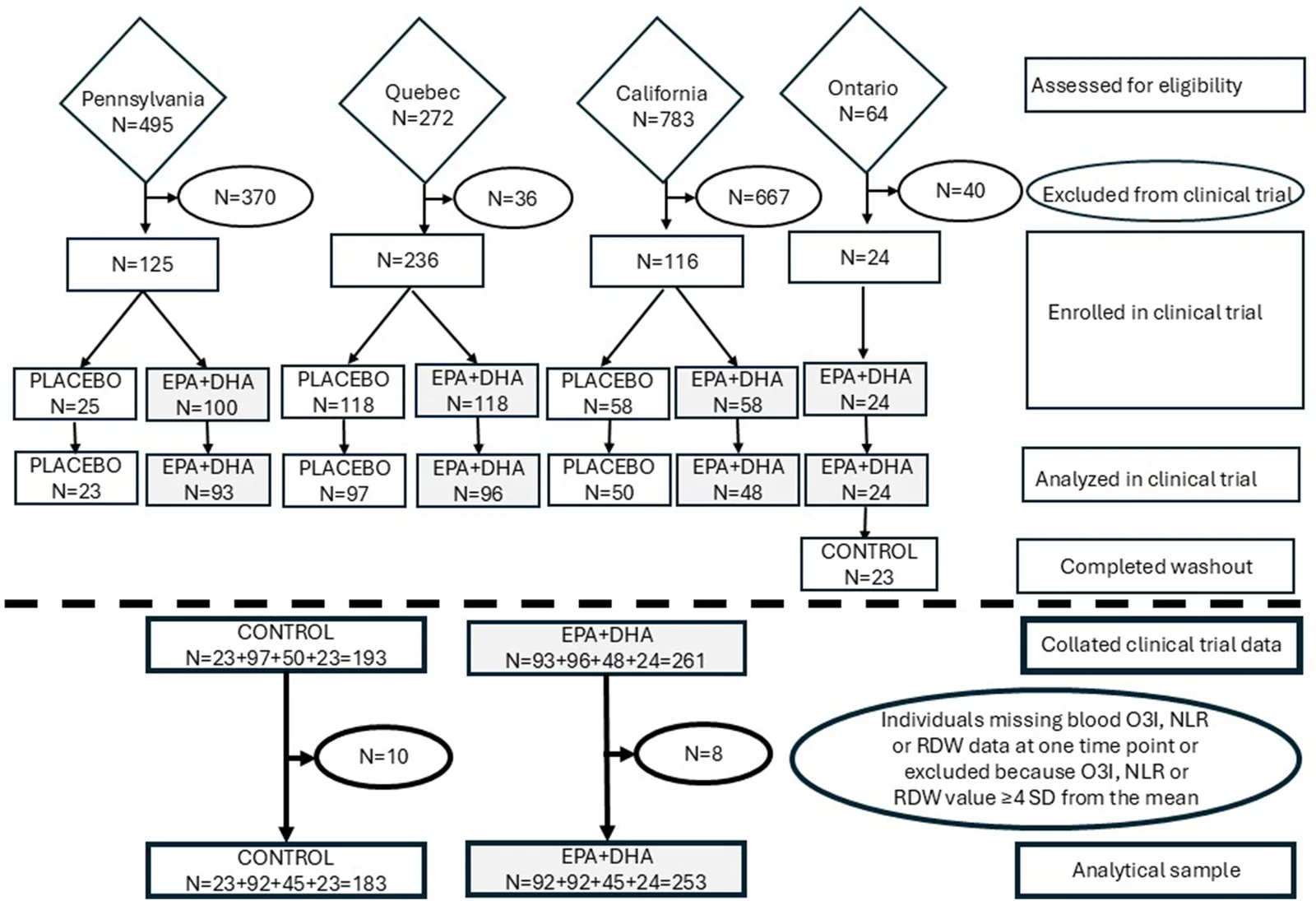
CONSORT diagram for individuals randomized and completing the 4 clinical trials (Pennsylvania, Quebec, California, and Ontario) and the collated clinical trial data (shown below the horizontal dotted line) with individual data for O3I, NLR, and RDW used for this analysis, where supplementation with EPA and DHAs is defined as EPA+DHA. Placebo or washout interventions are defined as CONTROL. NLR, neutrophil–lymphocyte ratio; O3I, Omega-3 Index; RDW, red blood cell distribution.

Four principal investigators with data from ω-3 fatty acid supplementation trials were approached and agreed to share anonymized individual-level data (treatment assignment (EPA+DHA or CONTROL), EPA+DHA dosage (mg/d), sex (male/female) and age (y), and b- and p-intervention O3I, NLR, RDW values) from 454 individuals who met study-specific baseline eligibility requirements and completed the clinical trials. The CONSORT diagram is presented (Figure 1). CONSORT diagrams for individual clinical trials are available in supplementary materials: Pennsylvania (Supplemental Figure 1), Quebec (Supplemental Figure 2), California (Supplemental Figure 3), and Ontario (Supplemental Figure 4). When O3I, NLR, or RDW values were missing for an individual at any sampling point, the individual was removed from further analysis, creating a final dataset of 436 individuals (Figure 1). The Research Ethics Board at the University of Guelph reviewed and approved this project, pooling anonymized individual-level data (EthOS Project ID: 1041; REB # 23-09-017).

### Omega-3 Index

Three studies (Pennsylvania, California, and Ontario) isolated RBCs from blood samples and analyzed their fatty acid content by GC (Table 1). The O3I is reported as EPA+DHA as a percentage of total identified RBC fatty acids [28]. The Quebec study extracted phospholipids from other plasma lipids, generated and analyzed fatty acid methyl esters by GC, and reported EPA and DHA as a percent of plasma phospholipid fatty acids [25]. For this dataset, RBC O3I values were calculated from the phospholipid EPA+DHA using the following formula: [(EPA%+DHA%) × 1.13] + 2.11, derived from a meta-regression analysis of 31 studies (*n* = 1701) and validated with *n* = 101 samples [29]. bO3I and pO3I were determined for each person, and the ΔO3I was calculated as pO3I minus bO3I.

### Hematologic indices

Blood samples were drawn by venipuncture, and N, L, and RDW values were determined by CBC tests using automated cell counters [25,26,30] (B. MacIntyre, M. Sterling, A. Jenkins, D.M. Mutch, unpublished results). For each individual, bNLR and pNLR were calculated as NLR = N/L, and ΔNLR was calculated as pNLR minus bNLR. Two NLR values that were >4 SD from the mean were removed from further analysis. RDW was treated the same as the NLR. Three RDW values that were >4 SD from the mean were removed from further analysis.

### Statistical analysis

Means/SDs and percentages/counts were used to summarize quantitative and categorical variables, respectively. Linear mixed effects models accounting for the repeated measures nature of the data were used to predict changes in primary outcome variables (O3I, NLR and RDW, separately), using a fixed effect for individuals assigned to ω-3 supplementation (EPA+DHA) compared with those assigned to placebo or washout (CONTROL), and a random effect for participant to account for the same participants occurring in both arms of the Ontario study (B. MacIntyre, M. Sterling, A. Jenkins, D.M. Mutch, unpublished results). Separate linear mixed effects models predicting the outcome were conducted for each of O3I, NLR, and RDW. Model 1 included a random effect for each person to predict the change in primary outcome, that is, O3I, NLR, or RDW, by treatment assignment (CONTROL or EPA+DHA). Model 2 further adjusted for bO31, bNLR, or baseline RDW (bRDW), respectively, as well as age and biological sex. A similar approach was taken using pooled ΔO3I, as the exposure in separate models predicting ΔNLR and ΔRDW. Model 1 adjusted for the random effects term, whereas model 2 adjusted for age, biological sex, and either bNLR or bRDW. A sensitivity analysis mirrored the primary analyses but dropped the data from the Ontario study because of its different design, that is, supplementation followed by washout. A significance level of 0.05 and 2-sided tests were used for all analyses.

## Results

### Pooled sample characteristics

Table 1 illustrates the characteristics of each study and the pooled sample (*N* = 436). The sample was ∼40% male, with an mean intervention duration of 18 wk, and a mean age of 38 y. Among the 436 participants, 253 were supplemented with at least 300 mg/d of EPA+DHA (mean = 1160 mg/d), whereas the remaining 183 participants assigned to placebo or washout (CONTROL) received 0 mg of EPA+DHA/d. For each of the 4 cohorts, bO3I, bNLR, and bRDW are presented in Table 2, respectively. By nature of the Ontario study design, that is, an EPA+DHA intervention followed by a washout, the mean CONTROL bO3I was higher (7.19) because it was the EPA+DHA pO3I. Similarly, the Ontario cohort CONTROL group had lower bNLR and bRDW. The California cohort of non-Hispanic Blacks had the lowest bNLR (Table 2), lowest bRDW, and greater variability than the other 3 studies.

**TABLE 2:** Baseline and postintervention ω-3 index, neutrophil–lymphocyte ratio, and red blood cell distribution width values by cohort and treatment.

Cohort	CONTROL		EPA+DHA	
	bO3I	pO3I	bO3I	pO3I
Pennsylvania	4.18 (0.92)	4.39 (0.95)	4.39 (1.16)	7.51 (1.80)
Quebec	4.96 (0.83)	4.31 (0.65)	5.13 (0.73)	8.93 (1.70)
California	4.03 (1.71)	3.88 (1.71)	3.90 (1.61)	6.33 (2.83)
Ontario	7.19 (1.27)^1^	4.96 (0.93)	3.70 (1.10)	7.22 (1.25)

	bNLR	pNLR	bNLR	pNLR

Pennsylvania	1.78 (0.69)	1.68 (0.57)	1.77 (0.62)	1.68 (0.57)
Quebec	1.73 (0.69)	1.72 (0.68)	1.71 (0.76)	1.72 (0.68)
California	1.36 (0.69)	1.31 (0.60)	1.53 (0.62)	1.31 (0.60)
Ontario	1.74 (0.78)^1^	1.85 (0.64)	1.83 (0.65)	1.85 (0.64)

	bRDW	pRDW	bRDW	pRDW

Pennsylvania	12.77 (0.51)	13.03 (0.46)	13.02 (0.66)	13.19 (0.68)
Quebec	13.16 (0.60)	13.20 (0.59)	13.10 (0.62)	13.15 (0.60)
California	11.83 (0.83)	11.87 (0.79)	12.02 (0.95)	11.90 (0.92)
Ontario	12.42 (0.60)^1^	12.66 (0.54)	12.55 (0.53)	12.46 (0.60)

Values are means (SD).

Abbreviations: bNLR, baseline neutrophil–lymphocyte ratio; bO3I, baseline Omega-3 Index; bRDW, baseline red blood cell distribution width; pNLR, postintervention neutrophil–lymphocyte ratio; pO3I, postintervention Omega-3 Index; pRDW, postintervention red blood cell distribution width.

1 Because of the nature of the Ontario study design (8 wk EPA+DHA supplementation followed by an 8-wk washout), the CONTROL bO3I mean is also the EPA+DHA supplementation pO3I mean.

### Changes in laboratory measurements by treatment assignment

**FIGURE 2:**
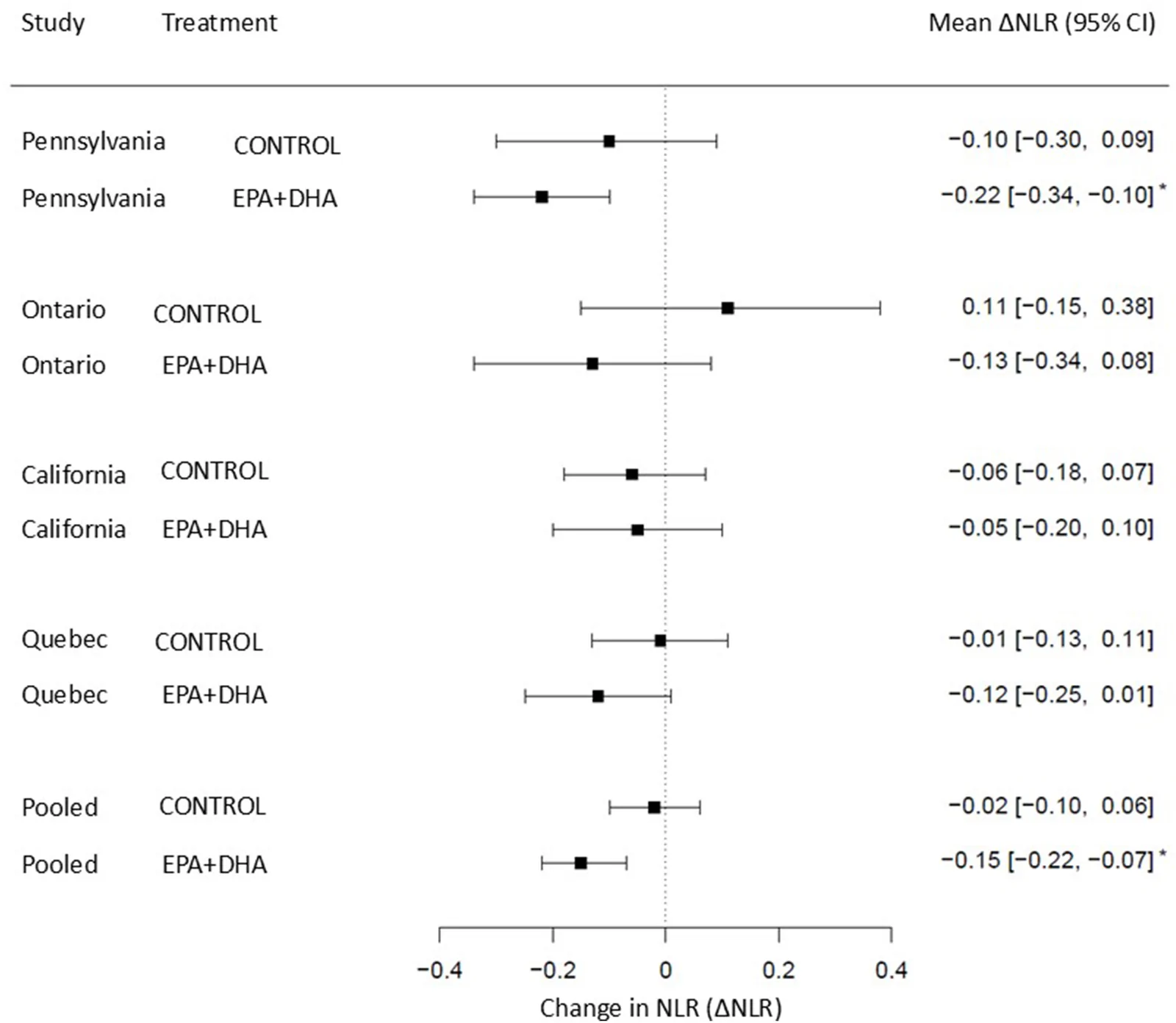
Forest plot of pooled individual ΔNLR by study and treatment. Treatments are supplementation with EPA+DHA or assignment to placebo or washout (CONTROL). ∗Signifies a statistical significance set at *P* < 0.05. CI, confidence interval; ΔNLR, changes in neutrophil–lymphocyte ratio.

Changes over time in the 3 outcomes of interest, that is, O3I, NLR, and RDW, by treatment are presented in Table 3. As expected, O3I increased with EPA+DHA supplementation, with an average ΔO3I = 3.28 (*P* < 0.001). In contrast, a smaller, but statistically significant, decrease in O3I was observed for individuals during CONTROL (mean ΔO3I = –0.62, *P* < 0.001). Hence, there was a net positive ΔO3I between the 2 interventions (ΔO3I = 3.94, *P* < 0.001) before (model 1) and after adjustment (model 2, ΔO3I = 3.84, *P* < 0.001). NLR decreased in the EPA+DHA group (ΔNLR = –0.15, *P* < 0.001), but there was no evidence of a statistically significant change in the CONTROL group (ΔNLR = –0.02, *P* ≥ 0.05) (Table 3). A statistically significant pooled treatment effect was observed for ΔNLR both before (model 1, ΔNLR = –0.13, *P* < 0.05) and after adjustment (model 2, ΔNLR = –0.10, *P* < 0.05). The pattern of results for ΔNLR was similar; that is, NLR decreased more in the EPA+DHA group across all cohorts except California (compared with CONTROL) (Figure 2). We observed a statistically significant 1% increase in RDW (ΔRDW = 0.09, *P* < 0.01) in the CONTROL group, but there was neither a statistically significant change in RDW with EPA+DHA supplementation, nor was there a pooled treatment effect before (model 1) or after (model 2) adjustment (Table 3).

**TABLE 3:** Baseline and postintervention means (SD) and changes in Omega-3 Index, neutrophil–lymphocyte ratio, and red blood cell distribution width using repeated measures analysis to account for the nature of the Ontario data^1^.

Treatment	Baseline mean (SD)	Post mean (SD)	Mean Δ (95% CI)^1^	Model 1 treatment effect (95% CI)^2^	Model 2 treatment effect (95% CI)^3^
	bO3I	pO3I			
CONTROL	4.91 (1.51)	4.30 (1.11)	–0.62 (–0.79, –0.44)∗∗∗	3.94 (3.63, 4.25)∗∗∗	3.84 (3.54, 4.13)∗∗∗
EPA+DHA	4.51 (1.23)	7.79 (2.16)	3.28 (3.04, 3.52)∗∗∗		
	bNLR	pNLR			

CONTROL	1.65 (0.72)	1.63 (0.67)	–0.02 (–0.10, 0.06)	–0.13 (–0.23, –0.02)∗	–0.10 (–0.19, –0.003)∗
EPA+DHA	1.71 (0.68)	1.57 (0.65)	–0.15 (–0.22, –0.07)∗∗∗		
	bRDW	pRDW			

CONTROL	12.70 (0.85)	12.83 (0.80)	0.09 (0.03, 0.15)∗∗	–0.04 (–0.12, 0.05)	–0.02 (–0.10, 0.06)
EPA+DHA	12.79 (0.83)	12.88 (0.85)	0.05 (–0.00, 0.11)		

Abbreviations: bNLR, baseline neutrophil–lymphocyte ratio; bO3I, baseline Omega-3 Index; bRDW, baseline red blood cell distribution width; Δ, changes; CI, confidence interval; pNLR, postintervention neutrophil–lymphocyte ratio; pO3I, postintervention Omega-3 Index; pRDW, postintervention red blood cell distribution width.

1 Significant changes are indicated as follows: ∗*P* < 0.05; ∗∗*P* < 0.01; ∗∗∗*P* < 0.001.

2 Linear mixed effects model. Model 1 included a random effect for each person to predict change in primary outcome, that is, O3I, NLR, or RDW, by treatment assignment.

3 Linear mixed effects model. Model 2 extended model 1 to further adjust for bO3I, bNLR, or bRDW (respectively), as well as age and sex.

### Correlations between changes in endpoints over time

**FIGURE 3:**
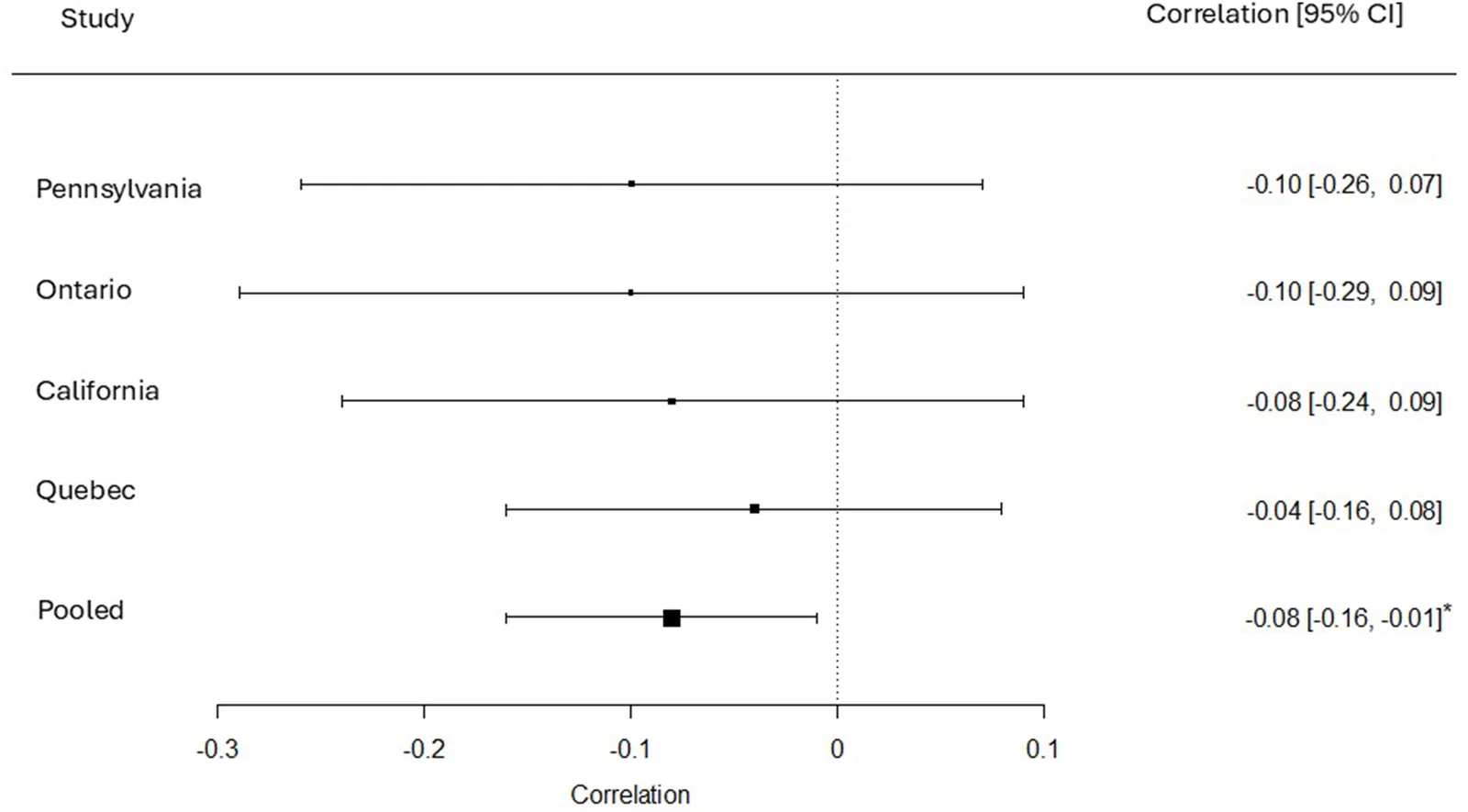
Forest plot of within-study and pooled-cohort correlations between ΔNLR and ΔO3I. ∗Signifies a statistical significance set at *P* < 0.05. CI, confidence interval; ΔNLR, changes in neutrophil–lymphocyte ratio; ΔO3I, changes in Omega-3 Index.

**FIGURE 4:**
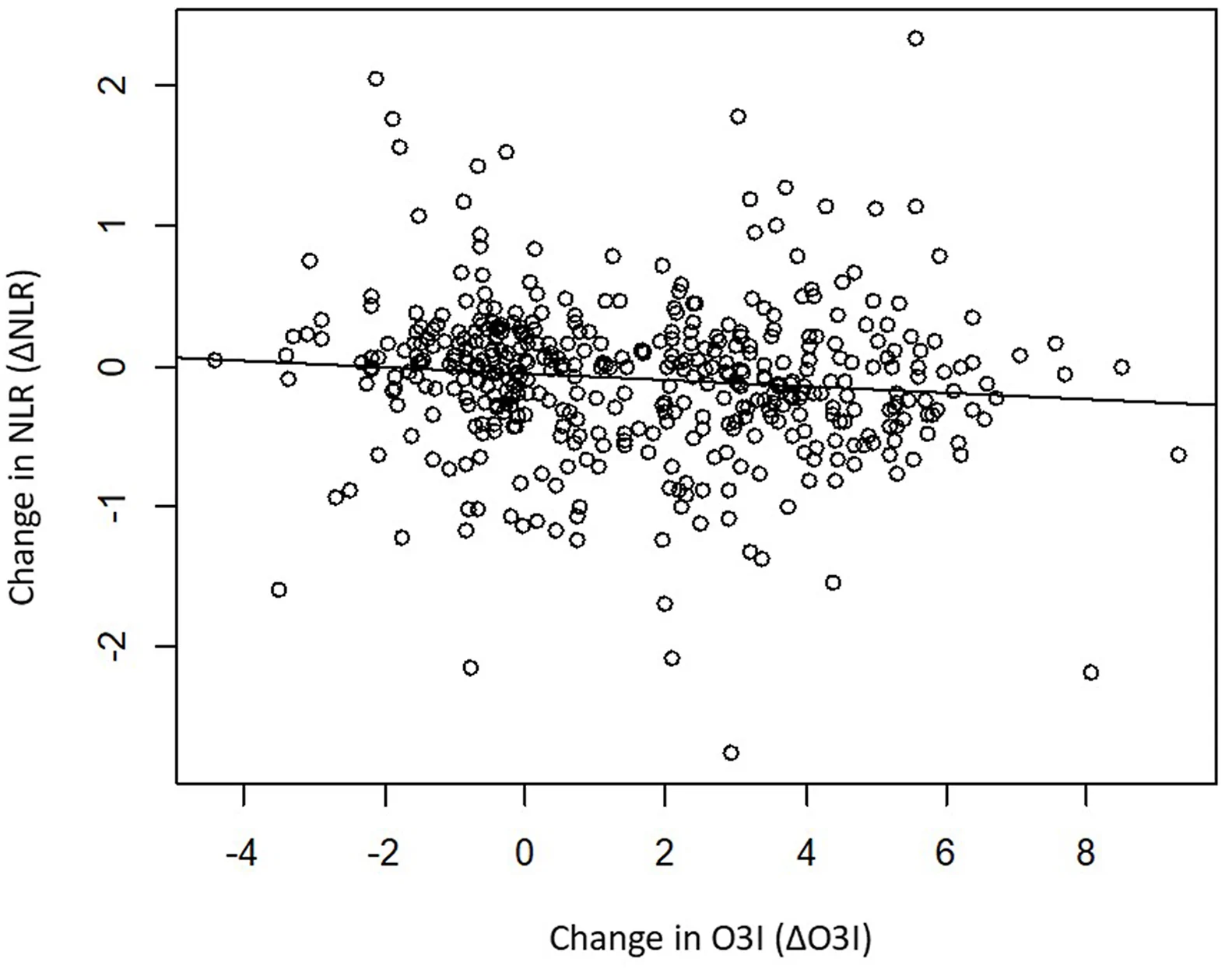
Scatterplot of the ΔNLR by ΔO3I (*r* = –0.08, 95% CI: –0.16, –0.01; *P* < 0.05). CI, confidence interval; NLR, neutrophil–lymphocyte ratio; ΔNLR, change in neutrophil–lymphocyte ratio; O3I, Omega-3 Index; ΔO3I, change in Omega-3 Index.

Next, we examined the correlations of ΔO3I with ΔNLR and ΔRDW, respectively. In pooled analyses, ΔNLR was inversely associated with the ΔO3I before (model 1, *r* = –0.08, *P* < 0.05) and after adjustment (model 2, *r* = –0.07, *P* < 0.05) (Table 4). All 4 cohorts showed inverse associations (Figure 3). A scatterplot of all individuals, along with a line of best fit, illustrated the inverse association between ΔNLR and ΔO3I (Figure 4). A nonstatistically significant correlation near 0 was observed between ΔRDW and ΔO3I in model 1 (*r* = 0.003), which remained nonstatistically significant after adjustment (model 2) (Table 4). A scatterplot of ΔRDW by ΔO3I for all individuals is shown in Supplemental Figure 5.

**TABLE 4:** Correlation between changes in O3I with changes in neutrophil–lymphocyte ratio and red blood cell distribution width using repeated measures analysis to account for the nature of the Ontario data^1^.

Variable	Correlation (95% CI)	
	Model 1^2^	Model 2^3^
ΔNLR	–0.08 (–0.16, –0.01)∗	–0.07 (–0.14, –0.002)∗
ΔRDW	0.003 (–0.09, 0.09)	0.04 (–0.05, 0.13)

Values are means (SD).

Abbreviations: bNLR, baseline neutrophil–lymphocyte ratio; bRDW, baseline red blood cell distribution width; CI, confidence interval; ΔNLR, change in neutrophil–lymphocyte ratio; O3I, Omega-3 Index; ΔRDW, change in red blood cell distribution width.

1 Significant changes are indicated as follows: ∗ *P* < 0.05.

2 Linear mixed effects model. Model 1 included a random effect for each person to predict the change in ΔNLR or ΔRDW by ΔO3I.

3 Linear mixed effects model. Model 2 extended model 1 and further adjusted for bNLR or bRDW, as well as age and sex.

### Sensitivity analysis

Because of the different design of the Ontario study, we restricted the data set to the 3 placebo-controlled RCTs and repeated the statistical analysis (Supplemental Table 1). O3I results were virtually unchanged. NLR point estimates were similar, but the treatment effect was no longer statistically significant. RDW point estimates were similar, and the treatment effect remained nonsignificant. The ΔNLR correlation with ΔO3I was similar (model 1 = –0.08, model 2 = –0.07) but no longer statistically significant. Finally, ΔRDW correlations with ΔO3I also remained nonsignificant (Supplemental Table 2).

## Discussion

### O3I results

This pooled cohort analysis confirms earlier reports [1,25,27,30] that EPA+DHA supplementation increases the O3I with a pooled ΔO3I = 3.28%. Our findings support the hypothesis that increased O3I reduced NLR (*P* < 0.05). RDW increased in CONTROL subjects (*P* < 0.001), but no changes in RDW were observed with EPA+DHA supplementation. ΔO3I was inversely correlated with NLR but not RDW. Participants recruited to these 4 clinical trials had bO3I ∼4.3%, that is, equivalent to a plasma EPA+DHA of 2.23% as reported for United States adults aged 20–55 y [31], which are somewhat lower than the O3I∼ 5.5% reported by [32] for the United States/Canada. The higher bO3I in the Ontario CONTROL group stemmed from the washout design, where the CONTROL bO3I was measured after 8 wk of supplementation with 2400 mg EPA+DHA/d. Observing considerable heterogeneity in individual responses to ω-3 supplementation, a secondary analysis of the California cohort reported ∼30% of the EPA+DHA group had habitual diets markedly low in green vegetable intake, which correlated with low O3I responses to ω-3 supplementation [33]. The reasons for this association are not clear. The lower pO3I in CONTROL individuals (*P* < 0.001) confirmed that participants followed guidance to minimize background (dietary) sources of ω-3 fatty acids during the study. Heterogeneity of individual responses to EPA+DHA supplementation, low dietary ω-3 intake when assigned to CONTROL interventions, and O3I decreasing from 7.19 to 4.96 during the 8-wk washout in the Ontario study contributed to greater ΔO3I variability and more power to assess the hypothesis that O3I causally affects NLR and RDW.

### NLR results

EPA+DHA supplementation decreased NLR (*P* < 0.0001). NLR did not change in CONTROL (*P* ≥ 0.05) despite a significant decrease in O3I (*P* < 0.001). In the pooled analysis, ΔO3I was inversely related to ΔNLR in these healthy adults. The lower bNLR observed for the California cohort was expected as non-Hispanic Blacks have, on average, significantly lower NLR compared with non-Hispanic Whites [9]. Our pooled cohort bNLR = 1.71 was lower than the average reported in population studies from the United States [NLR = 2.16; 95% confidence interval (CI): 2.13, 2.19] [34] and China (NLR = 1.77; 95% CI: 0.88, 4.0) [35]. This is consistent with the notion that people who volunteer for intervention studies are typically healthier than those screened in national surveys. Higher blood ω-3 concentration values have been associated with lower mortality in Framingham [36], Women’s Health Initiative Memory Study [37], and Fatty Acid and Outcome Research Consortium analysis [38]. Our findings that O3I was inversely correlated with NLR provide a biological rationale for observational findings in 7983 free-living adults aged ≥55 y where risk of death increased with each NLR quartile and the highest hazard ratio (HR; HR = 1.59; 95% CI: 1.37, 1.86) was found among individuals with NLR >2.41 [39]. In 46 individuals with moderate-to-severe head injury and elevated NLR (NLR = 10.08), randomly assigned to 300 mg EPA+DHA/d for 7 d, reduced NLR to 4.47 (compared with NLR = 7.39 in the placebo group, *P* = 0.015) [40]. In patients with stable coronary artery disease and bNLR ∼2.6, 3.36 g EPA+DHA/d for 30 mo reduced NLR (compared with placebo, *P* = 0.021) [8,40]. In conclusion, we provide further evidence that ω-3 supplementation increased O3I, decreased NLR, and ΔO3I was inversely related to ΔNLR, even in healthy individuals. Although statistically significant, the small reduction in NLR (ΔNLR = –0.15, *P* < 0.001) observed with ω-3 supplementation may not be clinically relevant in adults with bNLR <2.

### RDW results

RDW values have been positively correlated with risk of mortality in multiple observational studies involving public databases, typically by comparing groups with RDW <13.4% compared with RDW ≥13.4% [23]. A 1% higher RDW in patients admitted with congestive heart failure or in patients with coronary artery disease after discharge was associated with 11% and 18% increased risk of mortality, respectively [16]. We found that a decreased O3I (ΔO3I = –0.62, *P* < 0.001) was associated with a ∼1% increase in RDW in the CONTROL group (ΔRDW = 0.09, *P* < 0.01), but EPA+DHA supplementation was not associated with a significant change in RDW or ΔRDW. The inverse relationship between ΔO3I and ΔRDW in the CONTROL group is consistent with observational data where the O3I effect on RDW increased as O3I fell below 5.6% [6]. We did not observe a significant relationship between ΔO3I and ΔRDW in this healthy cohort. The high bRDW variability in the California cohort may have been a factor. RDW decreased from 14.8% to 13.8% (*P* = 0.003) in older adults (74 ± 5 y) with abdominal aortic aneurysm randomly assigned to 1.8 g EPA+DHA/d for 12 wk, whereas no change in RDW was observed with placebo [41]. Finally, RBCs circulate for 100–120 d (14–17 wk) [42], and 27% of individuals in the EPA+DHA group were supplemented for ≤8 wk. It may not be possible to reduce RDW in ω-3 interventions lasting <14 wk, especially in individuals with baseline RDW <12.7%.

Strengths of this pooled study are the availability of baseline and postintervention blood samples in each person, the diverse geographical and ethnic backgrounds, the range of ages of participants, the span of EPA+DHA doses, and the varying intervention durations. Some of these strengths, that is, varying EPA+DHA doses and intervention durations in the different cohorts, could be considered weaknesses, as could the fact that measurement of O3I, NLR, and RDW was not all conducted in the same laboratory for each study. However, differences between laboratories in O3I, NLR, and RDW become less important when baseline and postintervention samples were measured in the same laboratory. Our decision to include an ω-3 supplementation trial (Ontario cohort) with washout data, that is, where blood samples collected after 8-wk EPA+DHA also serve as CONTROL baseline O3I, NLR, and RDW measurements, along with the 3 placebo-controlled, parallel group, ω-3 supplementation trials, could also be considered a design weakness. However, the Ontario study permitted examination of the impact of larger reductions (–31%) in O3I over time (associated with 6% and 1.9% increased NLR and RDW, respectively) than could be obtained from placebo controls. Moreover, our individual cohort analyses showed the same patterns in the Ontario cohort as the other 3. Furthermore, a sensitivity analysis restricted to the 3 placebo-controlled RCTs found similar results, although less statistically significant due to the decreased sample size. Finally, heterogeneity in O3I responses to ω-3 supplementation, that is, as reported for the California cohort [33], contributes to the robustness of findings that ΔO3I correlates with ΔNLR.

In this pooled cohort of 4 ω-3 intervention studies involving healthy individuals, supplementation with ω-3 increased O3I (*P* < 0.001), decreased NLR (*P* < 0.05), and did not change RDW (*P* ≥ 0.05). In the CONTROL group, O3I decreased, NLR did not change, and RDW increased. Changes in O3I (ΔO3I) were inversely correlated with ΔNLR (*P* < 0.05) but not ΔRDW (*P* ≥ 0.05). Additional ω-3 intervention studies are needed in individuals with clinically elevated NLR, and possibly RDW, to determine whether EPA+DHA supplementation will improve these 2 hematological indices, which have been linked with multiple health outcomes.

## Author contributions

The authors’ responsibilities were as follows – MIM: conceived the hypothesis and objectives, identified collaborators with individual-level baseline and postintervention O3I, NLR, and RDW data from controlled ω-3 supplementation trials, and collated results from 4 clinical trials into a single dataset; NLT, JW: conducted the statistical analyses; MIM, NLT, WSH: drafted the first version of the manuscript; NLT: prepared the figures; MIM, NLT: prepared the tables; and all authors: contributed to the intellectual content, edited the manuscript, and read and approved the final version.

## Funding

Partial support for this project was received by a grant to the Fatty Acid Research Foundation from Myriad Canada. JN was partially supported by USDA Project 2032-10700-003-000-D. The USDA is an equal opportunity provider and employer.

## Declaration of generative AI and AI-assisted technologies in the writing process

The authors declare that no generative AI or AI-assisted technologies were used in the writing of this manuscript.

## Conflict of interest

The first author, MIM, has no conflicts of interest. The remaining authors have reviewed the manuscript and indicated they have not conflicts of interest.
