# Supplementary material for: Inverse Association between the Omega-3 Index and Neutrophil–Lymphocyte Ratio: Pooled Results from Four Supplementation Trials
Source: J Nutr. 2026 Jun 10;156(8):101663. doi: 10.1016/j.tjnut.2026.101663 (PMC13494177; doi:10.1016/j.tjnut.2026.101663)
Supplement: Supplementary file 1 [file mmc1.docx]

**Supplemental Figure 1:** Consolidated Standards Of Reporting Trials (CONSORT) diagram published for the Pennsylvania cohort(Flock et al. 2013b). NCT01078909

**Supplemental Figure 2:** Consolidated Standards Of Reporting Trials (CONSORT) diagram published for the Quebec cohort (Maltais et al. 2022). NCT1625195

**Supplemental Figure 3:** Consolidated Standards Of Reporting Trials (CONSORT) diagram published for the California cohort (Stephensen et al. 2011). NCT00536185

**Supplemental Figure 4:** Consolidated Standards Of Reporting Trials (CONSORT) diagram for the Ontario cohort (MacIntyre et al. unpublished).

**Supplemental Figure 5:** Scatterplot of the change in red blood cell distribution width (∆RDW) by change in omega-3 index (∆O3I) (r=0.003, 95% CI: -0.09, 0.09; p≥0.05).

| **Supplementary Table 1.** Baseline and post-intervention means (SD) and changes in omega-3 index, neutrophil-lymphocyte ratio, and red blood cell distribution width after DROPPING ONTARIO DATA^1,2^. | | | | | |
| --- | --- | --- | --- | --- | --- |
| **Treatment** | **Baseline**  **Mean (SD)** | **Post Mean (SD)** | **Mean ∆  (95% CI)^2^** | **Model 1**  **treatment effect (95% CI)^3^** | **Model 2**  **treatment effect**  **(95% CI)^4^** |
|  | **bO3I** | **pO3I** |  |  |  |
| CONTROL | 4.59 (1.23) | 4.20 (1.10) | ***-0.39 (-0.55, -0.22)****** | ***3.69 (3.35, 4.02)****** | ***3.76 (3.44 4.08)****** |
| EPA+DHA | 4.59 (1.21) | 7.85 (2.23) | ***3.26 (3.00, 3.51)****** |  |  |
|  |  |  |  |  |  |
|  | **bNLR** | **pNLR** |  |  |  |
| CONTROL | 1.63 (0.71) | 1.60 (0.67) | -0.04 (-0.12, 0.04) | ***-***0.11 (-0.22, 0.00) | -0.07 (-0.17, 0.03) |
| EPA+DHA | 1.70 (0.68) | 1.55 (0.63) | ***-0.15 (-0.22, -0.07)****** |  |  |
|  |  |  |  |  |  |
|  | **bRDW** | **pRDW** |  |  |  |
| CONTROL | 12.73 (0.88) | 12.81 (0.86) | ***0.07 (0.01, 0.13)**** | -0.00 (-0.09, 0.09) | 0.01 (-0.08, 0.10) |
| EPA+DHA | 12.85 (0.82) | 12.92 (0.86) | ***0.07 (0.01, 0.13)**** |  |  |
| ^1^ Abbreviations: baseline (b), post-intervention (p), omega-3 index (O3I), neutrophil-lymphocyte ratio (NLR), red blood cell distribution width (RDW), standard deviation (SD), changes in (∆).  ^2^ Significant changes are in **bold** and indicated as follows: *p<0.05; **p<0.01; ***p<0.001. | | | | | |
| ^3^ Linear mixed effects model. Model 1 included a random effect for each person to predict change in primary outcome, i.e., O3I, NLR or RDW, by treatment assignment. | | | | | |
| ^4^ Linear mixed effects model. Model 2, extended Model 1 to further adjust for bO3I, bNLR, or bRDW (respectively), as well as age and sex. | | | | | |

| **Supplementary Table 2.** Correlation between changes in omega-3 index (∆O3I) with changes in neutrophil-lymphocyte ratio and red blood cell distribution width after DROPPING ONTARIO DATA. Values are means (SD)^1,2^. | | |
| --- | --- | --- |
|  | **Correlation (r, 95% CI)** | |
| **Variable** | **Model 1**^3^ | **Model 2**^4^ |
| ∆NLR | -0.08 (-0.16, 0.01) | -0.07 (-0.14, 0.01) |
| ∆RDW | 0.07 (-0.03, 0.17) | 0.08 (-0.01, 0.18) |
| ^1^ Abbreviations: correlation coefficient (r), change in omega-3 index (∆O3I), change in neutrophil-lymphocyte ratio (∆NLR), change in red blood cell distribution width (∆RDW), standard deviation (SD).  ^2^ Significant changes are in **bold** and indicated as follows:^*^ p<0.05.  ^3^ Linear mixed effect model. Model 1 included a random effect for each person to predict change in ∆NLR or ∆RDW by ∆O3I. | | |
| ^4^ Linear mixed effects model. Model 2 extended Model 1 and further adjusted for for bNLR or bRDW, as well as age and sex. | | |
